# Novel symptoms associated with eclampsia could improve detection and save lives

**DOI:** 10.1371/journal.pmed.1005123

**Published:** 2026-05-21

**Authors:** Alice Beardmore-Gray, Andrew Shennan

**Affiliations:** Department of Women and Children’s Health, King’s College London, London, United Kingdom

## Abstract

Eclampsia is a life-threatening complication of pre-eclampsia, yet remains difficult to predict. In this Perspective, Alice Beardmore-Gray and Andrew Shennan highlight a recent study that identifies 10 novel prodromal symptoms of eclampsia, with potential to better predict which women are at risk and therefore reduce delays in intervention.

A woman dies every 2 min during pregnancy or childbirth [1]. Hypertensive disorders of pregnancy, including pre-eclampsia, are the leading direct cause after post-partum hemorrhage. Of these deaths, 95% occur in low- and middle-income countries (LMICs), and the regions of sub-Saharan Africa and South Asia account for 87% of all global maternal deaths [1]. This stark inequality is projected to deepen over the next decade, as funding scarcity, conflict, climate change, and a shifting geopolitical landscape threaten hard-won gains in women’s health [2].

Pre-eclampsia, a multi-system endothelial disorder caused by placental dysfunction, is progressive, and the onset of severe features is difficult to predict [3]. This is especially true in low-resource contexts where limited access to laboratory testing and obstetric ultrasound is further compounded by health workforce shortages, resulting in a lack of adequate surveillance for women at high-risk of complications and poor quality care [4].

Eclampsia is a manifestation of severe pre-eclampsia with an associated mortality rate of up to 7% [5]. It is frequently associated with additional complications including intracerebral hemorrhage, pulmonary oedema, placental abruption, and stillbirth. Current management includes administration of magnesium sulfate, which more than halves the risk of eclampsia and is indicated for both primary prevention and recurrence. It should be given to all women who have severe pre-eclampsia or eclampsia, whether antenatal or postnatal, and is recognized by the World Health Organization and United Nations to be a priority drug. While its exact mechanism of action is unknown, it is thought to prevent and control seizures by stabilizing neuronal networks and reducing cerebral vasospasm and ischemia.

Understanding which women with pre-eclampsia are most at risk of progressing to eclampsia and other severe complications is critical to informing women’s and clinicians’ decision-making and guiding clinical management. While stabilizing blood pressure is important, delivery is currently the only intervention shown to reduce the risk of adverse maternal outcomes [6] and fetal death [7], and should be recommended immediately if severe features, including eclampsia, develop [8]. Accurately triaging women to identify those most at risk, in order to optimize timing of birth, with consideration of antenatal corticosteroids if delivery is planned prior to 34 weeks’ gestation, remains a critical challenge.

Although there have been advances in the prediction and diagnosis of pre-eclampsia, there are still no prognostic tools that reliably predict the onset of complications once pre-eclampsia has been diagnosed. Moreover, clinical prediction tools that rely on laboratory tests, sonographer expertise, and biomarkers may be challenging and costly to implement in already fragile healthcare systems within LMICs. Clinical decision-making around ongoing management is therefore often based on the presence or absence of poorly defined “severe maternal symptoms” [8,9], which have limited prediction. This is confounded by clinical signs also being unreliable: A 2018 study demonstrated that high blood pressure was not significantly associated with eclampsia in an LMIC country (South Africa) [10], yet it is often used to define the severity of pre-eclampsia, particularly in low-resource settings. A woman with severe pre-eclampsia may need to be transferred from remote and rural settings in LMICs without diagnostic tools to a higher-level care facility in order to access life-saving interventions [11].

Thus, better prognostic tools are needed. The process of history taking has been described as “the most powerful and sensitive and most versatile instrument available to the physician” and has been reported to provide 60%–80% of the information that is relevant for a diagnosis [12]. Midwives and obstetricians have traditionally been taught to ask women with pre-eclampsia if they have a headache, visual changes, and/or epigastric pain as part of their clinical history and routine symptoms enquiry. However, these previously identified prodromal symptoms, based on a small number of methodologically limited studies, have shown only modest associations with eclampsia and do not accurately predict its onset, nor reliably rule it out if absent [13].

In a recent *PLOS Medicine* study [14], Hastie and colleagues have identified 10 novel prodromal symptoms exhibiting far stronger associations with eclampsia than the traditionally associated symptoms of headache, visual changes, and epigastric pain (Fig 1). This case-control study prospectively enrolled women over a 5-year period, with eclampsia, pre-eclampsia or normotensive pregnancies in South Africa and Pakistan and asked whether they experienced 20 neurological symptoms. For those women who experienced eclampsia (*n* = 341), this was within 7 days of the seizure. They compared the likelihood of symptoms occurring before eclampsia, compared to being present with pre-eclampsia. Ten symptoms had odds ratios (ORs) over 10 for eclampsia (whereas ORs for existing symptoms are all lower than 10: headache (OR 2.26), visual changes (OR 5.73), and epigastric pain (OR 2.25)). The newly identified prodromal symptoms are summarized in Fig 1.

**Fig 1:**
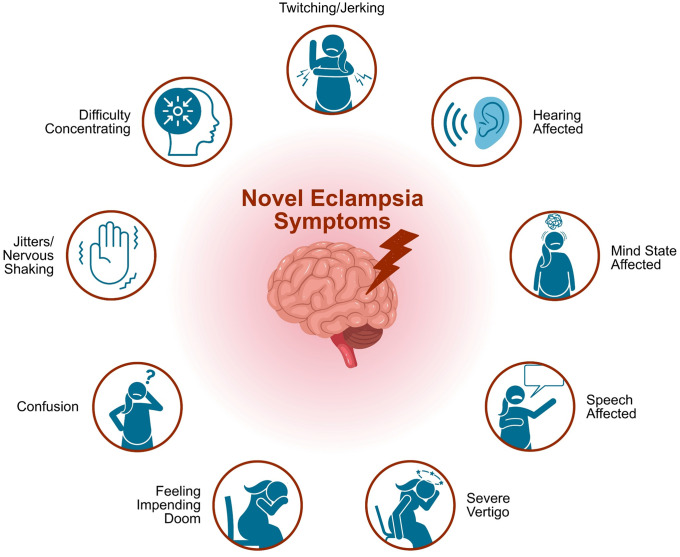
Ten novel prodromal symptoms of eclampsia. Novel symptoms (as identified in [14]) and their odds ratios (ORs) include: twitching/jerking limbs (OR 42.03), affected hearing (OR 33.12), altered mind state (OR 33.60), impaired speech (OR 33.12), feelings of doom (OR 23.71), severe vertigo (OR 26.59), confusion (20.52), jitters (OR 18.16), difficulty concentrating (OR 15.81), and weakness/paralysis (OR 10.49).

Notably, only 2.4% of women who experienced eclampsia did not report any of the full list of 20 prodromal symptoms screened for in this study, highlighting the importance of continually re-assessing women’s symptoms. Furthermore, those who had eclampsia were younger, had a lower Body Mass Index, were more likely to be nulliparous, book later for antenatal care, or have not received any antenatal care. This represents a high-risk group of women who require heightened surveillance and pro-active inclusion within healthcare systems.

This study is strengthened by its sites in two countries which represent regions of the world with a high burden of pre-eclampsia and maternal death, thus increasing the generalizability of their findings across different populations and healthcare systems. Eclampsia, while serious, is a relatively rare complication, and therefore to have evaluated a cohort of 341 women who experienced eclampsia is impressive and increases the robustness of their findings. Expert neurology input into this study also highlights the importance of integrating obstetric and medical teams as part of providing holistic care and developing a broad understanding of this multi-system disorder, which also increases future risk of cardiovascular and cerebrovascular disease.

While it is possible that women affected by eclampsia may have recalled their symptoms differently, the study investigators were able to mitigate the risk of recall bias by ensuring that the entire symptoms list was screened for within all groups, including women with pre-eclampsia and normotensive pregnancies. Moreover, given the rare occurrence of eclampsia, a prospective screening study would likely not have been feasible.

The proportion of women who did not receive antenatal care differed between the two study sites (6.6% in South Africa versus 43.4% in Pakistan). Future work should focus on evaluating these symptoms in an even larger cohort of women across multiple different care settings, to establish whether screening for these prodromal symptoms is similar in all settings. Correlation of symptom severity with blood pressure and other markers of pre-eclampsia severity (biochemistry, ultrasound findings, placental growth factor levels) could also add further information to a future predictive model.

While acknowledging that this was not intended to be a diagnostic prediction study, and that further prospective validation of these symptoms’ predictive ability is warranted, maternity care providers should implement screening for these 10 symptoms identified as being strongly associated with eclampsia into clinical protocols. It is important that educators and policymakers take these findings into account when updating training curriculums and guidelines, and that further research evaluates effective dissemination and implementation of such materials.

Women’s health remains neglected, under-funded, and under-prioritized, and this contributes to poor reproductive health outcomes for women. Women spend nearly 25% more of their lives in poor health than men, yet less than 5% of global health research funding is directed toward women-specific conditions beyond oncology. This study therefore offers a timely and relevant contribution to the existing evidence, questioning previous practice and expanding our understanding of a condition which remains a leading cause of maternal death globally. The identification of these 10 prodromal symptoms, which may predict the onset of eclampsia, offers real-world clinical utility and has the potential to offer a simple, low-cost screening tool that could prompt earlier intervention and thus reduce maternal and perinatal mortality.

In an era increasingly dominated by high-tech solutions—artificial intelligence, digital health, and precision medicine—this study reminds us that the most transformative interventions are often the simplest. A better history, grounded in robust evidence, costs nothing and can be implemented anywhere. We look forward to seeing how these findings will be taken forwards and ultimately incorporated into routine clinical practice.

## Abbreviations

LMICs: low- and middle-income countries
ORs: odds‌‌ ratios
